# Cryopreserved Mesenchymal Stromal Cells Maintain Potency in a Retinal Ischemia/Reperfusion Injury Model: Toward an off-the-shelf Therapy

**DOI:** 10.1038/srep26463

**Published:** 2016-05-23

**Authors:** Oliver W. Gramlich, Anthony J. Burand, Alex J. Brown, Riley J. Deutsch, Markus H. Kuehn, James A. Ankrum

**Affiliations:** 1 Department of Ophthalmology and Visual Sciences, University of Iowa, Iowa City, IA, USA; 2 Center for the Prevention and Treatment of Visual Loss, Iowa City VA Health Care, Iowa City, IA, USA; 3 Department of Biomedical Engineering, University of Iowa, Iowa City, IA, USA; 4 Fraternal Order of Eagles Diabetes Research Center, Pappajohn Biomedical Institute, University of Iowa, Iowa City, IA, USA.

## Abstract

The ability to use mesenchymal stromal cells (MSC) directly out of cryostorage would significantly reduce the logistics of MSC therapy by allowing on-site cryostorage of therapeutic doses of MSC at hospitals and clinics. Such a paradigm would be especially advantageous for the treatment of acute conditions such as stroke and myocardial infarction, which are likely to require treatment within hours after ischemic onset. Recently, several reports have emerged that suggest MSC viability and potency are damaged by cryopreservation. Herein we examine the effect of cryopreservation on human MSC viability, immunomodulatory potency, growth factor secretion, and performance in an ischemia/reperfusion injury model. Using modifications of established cryopreservation methods we developed MSC that retain >95% viability upon thawing, remain responsive to inflammatory signals, and are able to suppress activated human peripheral blood mononuclear cells. Most importantly, when injected into the eyes of mice 3 hours after the onset of ischemia and 2 hours after the onset of reperfusion, cryopreserved performed as well as fresh MSC to rescue retinal ganglion cells. Thus, our data suggests when viability is maintained throughout the cryopreservation process, MSC retain their therapeutic potency in both *in vitro* potency assays and an *in vivo* ischemia/reperfusion model.

Mesenchymal stromal/stem cells (MSC) have been explored in hundreds of clinical trials for the treatment of dozens of conditions^1^,^2^. While MSC can be harvested from nearly any tissue^3^, they are a rare cell type^4^ and thus typically require significant *ex vivo* expansion to generate therapeutic doses of cells. Allogeneic MSC are used in most clinical trials as MSC are immune evasive, allowing them to avoid immediate immune detection and clearance^2^. Allogeneic MSC are typically expanded in culture, cryopreserved, and banked for future use, creating the opportunity for an ‘off-the-shelf’ therapy.

Many proposed applications of MSC therapy would require on demand access to therapeutic doses of MSC and therefore necessitate access to cryopreserved MSC stocks. Acute conditions including acute graft versus host disease (GvHD), acute kidney injury, acute lung injury, and sudden onset ischemic events such as myocardial infarction, acute limb ischemia, retinal and optic neuropathies, and stroke would all benefit from rapid MSC administration within hours after the onset of symptoms. The mechanism of action of MSC in these conditions is thought to be mediated through both modulation of inflammatory reactions as well as secretion of protective growth factors^5^. Even if a disease indication could accommodate a post-thaw recovery period ranging from hours to days, logistically, use of MSC immediately post-thaw would still be preferable, since post-thaw recovery needs to be carried out by experienced technicians in dedicated facilities. This not only leads to quality control issues but also adds significant infrastructure requirements that will prevent the use of MSC therapies in many hospitals. Therefore, identification of conditions that preserve MSC function throughout cryopreservation as well as disease indications that allow MSC to be applied directly post-thaw is critical to the development of truly ‘off-the-shelf’ MSC therapies.

Although multiple groups have investigated the impact of cryopreservation on the phenotype of MSC, studies to date have yielded conflicting results and many questions remain. Most importantly, do changes in phenotype caused by cryopreservation have a meaningful impact on therapeutic efficacy? Luetzkendorf *et al.* examined changes in MSC proliferation, viability, and immunosuppressive potential after cryopreservation^6^. In this study cryopreserved MSC showed no difference in proliferation or viability post-thaw. When co-cultured with PHA-stimulated peripheral blood mononuclear cells (PBMC), MSC’ immunosuppressive potency after thaw varied depending on MSC donor. Two donors exhibiting enhanced suppression after cryopreservation, one donor exhibited reduced potency, and a fourth donor had highly variable function^6^. Galipeau and colleagues recently reported freshly thawed MSC exhibit significantly diminished viability compared to cells that had been in culture for greater than 7 days^7^. In addition, freshly thawed MSC showed reduced response to interferon-γ (IFN-γ). Notably, maintenance in culture for 7 days restored MSC sensitivity to IFN-γ and indoleamine 2,3-dioxygenase (IDO) expression, suggesting the observed impairment was transient. The reduced viability and expression of immunomodulatory factors in freshly thawed MSC also resulted in reduced suppression of activated T-cells and, in some cases, actually led to hyper-proliferation of T-cells in co-culture assays. The authors hypothesized that these phenomena are due to the presence of large numbers of dead cells^7^. The same group subsequently reported that the actin cytoskeleton of freshly thawed MSC is disrupted, leading to reduced adhesion to endothelium and poor engraftment following intravenous infusion. Again, recovery in culture for 48 hours restored this aspect of MSC function^8^. Moll *et al.* recently compared the propensity of freshly thawed MSC to activate the complement cascade and induce an instant blood mediated inflammatory reaction (IBMIR)^9^. In their study, freshly thawed MSC were more susceptible to destruction by IBMIR and complement activation. They also demonstrated that freshly thawed MSC had lower levels of IDO transcripts after IFN-γ stimulation and had diminished immunosuppressive potency in co-cultures with activated PBMCs isolated from whole blood. In contrast to the Galipeau paper^7^ and in agreement with the Leutzkendorf paper^6^, the viability of freshly thawed MSC was observed to be similar to the viability of MSC harvested from continuous cultures, likely a consequence of differences in MSC donors and/or cryopreservation/thaw procedures used in the respective labs. In addition to *in vitro* analysis, Moll *et al.* also compared the clinical response of acute GvHD patients receiving intravenous injections of thawed MSC versus MSC collected from continuous cultures. Overall patients receiving fresh MSC, particularly early passage fresh MSC, had a much improved clinical response compared to patients receiving cryopreserved MSC^9^.

Herein we seek to determine the effect of cryopreservation on human MSC’s suitability to treat acute ischemic and inflammatory conditions. MSC phenotype, including viability, growth potential, growth factor secretion, expression of immunomodulatory factors, and ability to suppress activated inflammatory cells are analyzed in passage and donor matched MSC with and without cryopreservation. Finally, we test an ‘off-the-shelf’ MSC therapy treatment paradigm in a retinal ischemia/reperfusion injury model designed to replicate a clinical scenario.

## Results

### Cryopreservation marginally impairs MSC viability and metabolic activity

**Figure 1:**
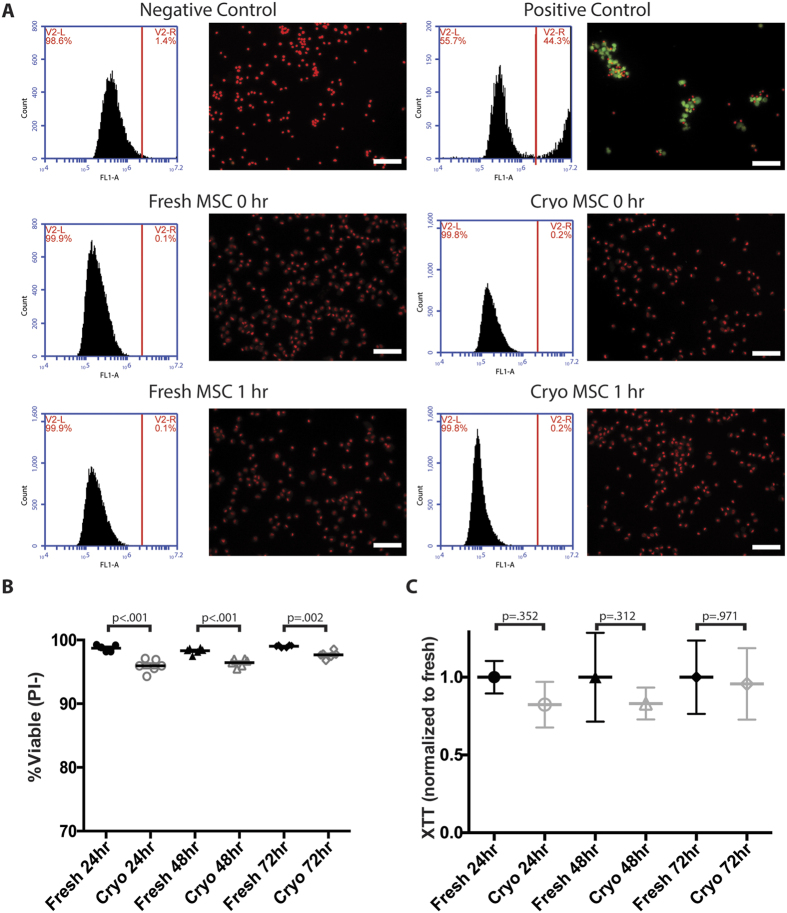
Cryopreservation marginally affects MSC viability and metabolic activity. (**A**) MSC harvested from culture or thawed directly out of cryostorage were assayed for double strand DNA breaks by TUNEL staining. MSC were analyzed immediately after thawing or after 1 hour of storage on wet ice by flow cytometry and fluorescence imaging. The percent of positive and negative stained cells is reported in the upper right and left corners of each plot respectively. Cells were fixed, stained with PI (red) and FITC-dUTP (green). Double positive cells were considered dead. (Scale Bar = 100 μm). (**B**) Viability of MSC plated after thawing was compared to donor and passage matched MSC from fresh cultures 24, 48, and 72 hours after thawing. Cells were stained with Hoechst 33342 and PI and imaged with a fluorescence microscope. Cells double positive for Hoechst 33342 and PI were considered dead. (One-way ANOVA with Sidak correction for multiple comparisons, p < 0.05 considered significant, n = 5). (**C**) The metabolic activity of MSC after cryopreservation was compared to donor and passage matched MSC from continuous cultures using XTT (mean ± SD, One-way ANOVA with Sidak correction for multiple comparisons, p < 0.05 considered significant, n = 6). All experiments performed with MSC from donors 8002L and 7083 at passages P3–P5.

Cryopreservation is an inherently stressful process for cells and it is not surprising to see detrimental effects on the viability and growth kinetics of cells immediately after thawing. Viability of MSC after thawing has been one of the most variable metrics in recent papers examining the use of cryo-MSC, ranging from as low as 50%^7^ to greater than 90% viability^6^,^9^. These disparate finding could be related to the fact that pores form in membranes following exposure to DMSO^10^ that could lead to false staining with traditional cell death markers including PI and Annexin V. Thus, here we sought to characterize the viability and growth kinetics of MSC following cryopreservation by directly labeling double stranded DNA breaks characteristic of cell death in MSC in the hour immediately post-thaw. In addition, we measured viability and metabolic activity 24, 48, and 72 hours after thawing to determine if cryopreservation has any lasting impact on MSC. Staining for double strand breaks with TUNEL immediately after thawing and after 1 hour of storage on ice revealed that MSC viability is not significantly reduced by cryopreservation when carried out as described herein (0.1–0.2% of cells stained positive by TUNEL in all groups, Fig. 1A). In contrast, when viability was assessed by PI staining cryo-MSC displayed a minor, but statistically significant, reduction in viability both 24 and 48 hours after thawing (2.8% and 1.9% reduction respectively, n = 5, p < .05, Fig. 1B). Differences in viability were no longer significant 72 hours after thawing (1.3% reduction, Fig. 1B). Similarly, while cryo-MSC displayed slightly lower metabolic activity than fresh MSC at 24 (18% lower), 48 (17% lower), and 72 hours (4% lower) after thawing, as measured by XTT, differences were not statistically significant at any time point (Fig. 1C). Overall, cryopreservation and cell handling, as performed in this study, appears to only marginally reduce MSC viability and metabolic activity.

### Cryopreserved MSC Maintain Immunomodulatory Potential

Sterile inflammation following ischemia/reperfusion injury leads to the destruction of cells in the tissue that would otherwise survive the ischemic insult. MSC can prevent this untargeted damage by inducing neutrophil apoptosis through the expression of IDO, which produces kynurenine metabolites known to be toxic to neutrophils^11^,^12^. In addition, MSC express a variety of factors that have been demonstrated to directly suppress T-cell activation and proliferation^13^. Thus, we sought to determine if MSC’s immunomodulatory potency was impaired during cryopreservation.

**Figure 2:**
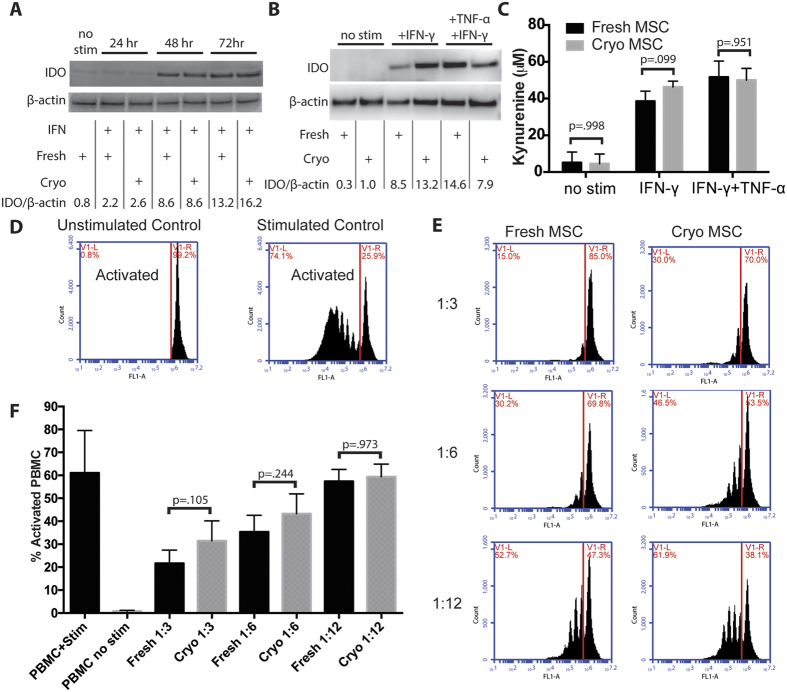
Cryopreserved MSC maintain immunosuppressive potential. (**A**) Representative Western blot of IDO protein in fresh and cryo-MSC after exposure to IFN-γ for 24, 48, or 72 hours. β-actin provided as a loading control. (**B**) Representative Western blot of IDO in fresh and cryo-MSC after exposure to IFN-γ or TNF-α/IFN-γ for 48 hours. β-actin provided as a loading control. (**C**) IDO activity as measured by the concentration of kynurenine in the conditioned media collected from fresh or cryo-MSC exposed to IFN-γ or TNF-α/IFN-γ for 48 hours (mean ± SD, One-way ANOVA with Sidak correction for multiple comparisons, p < 0.05 considered significant, n = 6). (**D**) Example unstimulated and stimulated PBMC controls used for gating and setting the activation threshold. PBMCs stained with CFSE remain as a single population in unstimulated conditions but upon stimulation with CD3/CD28 dynabeads become activated and proliferate. (**E**) Example flow cytometry histograms of stimulated CFSE stained PBMCs co-cultured with fresh or cryo-MSC at MSC:PBMC ratios of 1:3, 1:6 or 1:12. (**F**) Quantification of the percent of activated PBMCs in each co-culture condition compared to unstimulated and stimulated controls. No statistical differences between fresh MSC and cryo-MSC at each ratio. (mean ± SD, One-way ANOVA with Sidak correction for multiple comparisons, p < 0.05 considered significant, 1:3 and 1:6 n = 4, 1:12 n = 3). All experiments performed with MSC from donors 8002L and 7083 at passages P3–P5.

We have previously described that IDO expression varies significantly by MSC donor and passage^14^, and consequently all experiments here were completed with donor and passage matched MSC. MSC exposed to IFN-γ immediately after thawing expressed similar levels of IDO as MSC maintained in fresh cultures (Fig. 2A). Very little IDO was detectable in either fresh or cryo-MSC after 24 hours of cytokine stimulation, but IDO levels increased similarly in both groups 48 and 72 hours after stimulation. In addition, both fresh and cryo-MSC stimulated for 48 hours with either IFN-γ or IFN-γ and TNF-α displayed high levels of IDO protein expression (Fig. 2B) and concomitant IDO activity as measured by kynurenine production (Fig. 2C). Next, to determine if cryo-MSC maintain their ability to suppress T-cell activation, we performed a co-culture experiment with primary human PBMCs. Unstimulated PBMCs and PBMCs stimulated with CD3/CD28 dynabeads served as unactivated and activated controls, respectively (Fig. 2D). Both fresh and cryo-MSC were able to suppress proliferation of PBMCs when cultured at MSC:PBMC ratios of 1:3, 1:6 and 1:12 (Fig. 2E). Mean PBMC proliferation rates in the presence of fresh MSC were 21%, 35%, and 57%, respectively, whereas PBMC in the presence of cryo-MSC proliferated at 31%, 43%, and 59%, respectively (Fig. 2F). None of the differences were statistically significant (1:3 and 1:6 n = 4, 1:12 n = 3). Thus, in our hands, cryopreservation did not significantly impair MSC’s immunomodulatory potential.

### Effect of Cryopreservation on MSC Secretome

**Figure 3:**
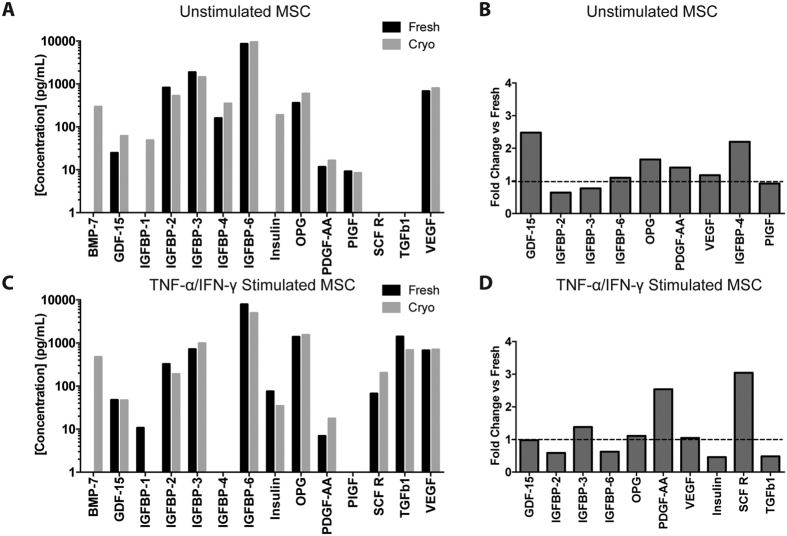
Cryopreservation minimally impacts baseline or stimulated growth factor secretion by MSC. Fresh and cryo-MSC, both passage 4 MSC from donor 7083, were cultured for 48 hours in reduced serum media with or without stimulation by TNF-α/IFN-γ, the media was collected, and screened for 40 growth factors. Of the 40 proteins, 14 were detectable in at least 2 of the tested conditions and are reported here. Values represent measured concentration after background subtraction (unconditioned control media). A full table of values is available in Supplemental Figure 1. (**A**) The concentration of each growth factor produced by fresh and cryo-MSC over 48 hours in unstimulated conditions. (**B**) Fold change in concentration in unstimulated MSC, [Cryo]/[Fresh] (**C**): The concentration of each growth factor produced by fresh and cryo-MSC over 48 hours in stimulated conditions. (**D**) Fold change in concentration in stimulated MSC, [Cryo]/[Fresh]. Dashed line at 1 corresponds to no-change between groups.

In addition to blunting a T-cell mediated inflammatory response, the ability of MSC to support cell survival in ischemia/reperfusion could also involve the synthesis of growth factors that prevent cell death and aid the reestablishment of the vasculature. Indeed, VEGF secreted from MSC has previously been shown to reduce neuronal loss in a rat stroke model^15^ and PDGF secreted from MSC has been implicated as being neuroprotective for retinal ganglion cells^16^. Thus, we sought to identify any potential changes in the MSC growth factor secretome that arises due to cryopreservation. The quantity and composition of the MSC secretome is heavily dependent on MSC donor and over 10-fold differences in expression and secretion levels have been observed by multiple groups when comparing individual donors subjected to identical culture conditions^14^,^17^,^18^,^19^. Thus, we analyzed fresh and cryopreserved passage matched MSC derived from a single donor, #7083, in order to isolate the impact of cryopreservation on the MSC secretome. Fresh or cryo-MSC, from donor #7083, were plated in reduced serum (1% FBS) growth media with or without a cytokine cocktail selected to mimic *in vivo* inflammatory conditions. After 48 hours, the media was collected and analyzed for the presence of 40 growth factors. A complete table of growth factor concentrations for fresh and cryo-MSC with and without cytokine stimulation is provided as Supplemental Table 1. Of the 40 screened factors, only 14 were detectable in at least 2 of the 4 conditions tested, and their concentrations are displayed in Fig. 3A,C. In addition, as a statistical measure of the impact of cryopreservation on the production of each growth factor, the effect size, displayed as fold change compared to fresh MSC is plotted in Fig. 3B,D. Overall, the profile and magnitude of growth factor expression was similar when comparing cryo-MSC to fresh MSC in both the unstimulated (Fig. 3A,B) and stimulated conditions (Fig. 3C,D). Expression of PDGF which is known to be anti-apoptotic and pro-angiogenic, was slightly elevated in cryo-MSC compared to the fresh MSC while VEGF levels remained similar in both groups. Cytokine stimulation resulted in marked increase in secretion of both stem cell factor receptor (SCF R/c-kit) and TGF-β1 in both fresh and cryo-MSC. BMP-7 exhibited the most striking difference between cryo-MSC and fresh MSC. It was robustly expressed in both unstimulated and stimulated cryo-MSC, but was undetectable in both forms of fresh MSC. Overall, differences in secreted factors between fresh and cryo-MSC were subtle and the impact of these differences must be considered in the context of specific therapeutic applications. Thus, we turned to an *in vivo* model of retinal ischemia reperfusion injury to determine if MSC’s therapeutic potency was impaired by cryopreservation.

### Fresh MSC and cryo-MSC equally rescue retinal ganglion cells following I/R injury

**Figure 4:**
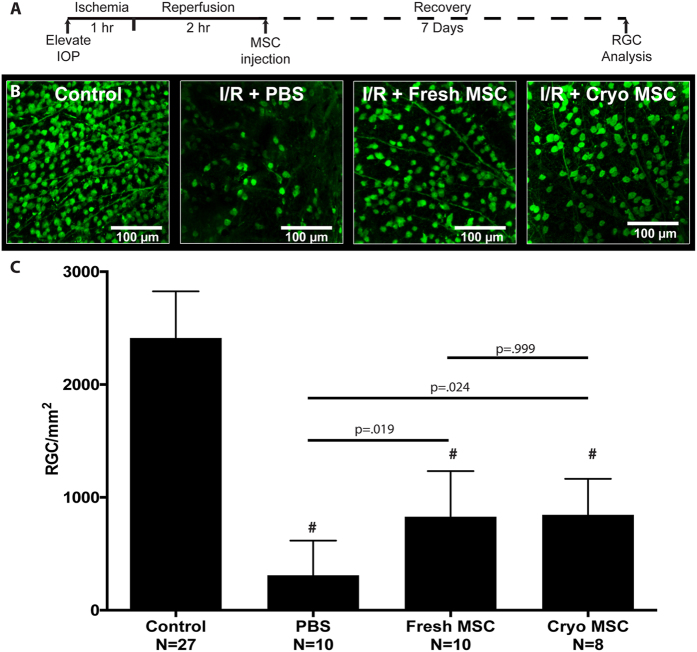
Cryopreserved MSC prevent RGC loss after ischemia/reperfusion injury *in vivo*. (**A**) Injury and treatment timeline for all retinal ischemia/reperfusion model. Intraocular pressure (IOP) was elevated to blanch the fundus for 1 hour, after which perfusion was restored. 2 hours after reperfusion, eyes were injected with one of the MSC groups or PBS as a vehicle control. 7 days later, animals were sacrificed and eyes were analyzed for RGC counts. (**B**) Representative images of γ-synuclein immunostaining of whole-mounted retina from non-ischemic retinas (control) and retinas after I/R treated with vehicle (I/R + PBS), fresh MSC (I/R + Fresh MSC) or cryo-MSC (I/R + Cryo-MSC). (**C**) Quantitative analysis of RGC survival in eyes after I/R revealed a significant rescue effect after transplantation of both fresh MSC and cryo-MSC (mean ± SD, One-way ANOVA with Tukey honest significant difference post hoc test to correct for multiple comparisons, p < 0.05 considered significant). #denotes p < 0.001 in comparison to healthy control. Both cryo-MSC and fresh MSC were from donor 7083.

In order to assess whether cryo-MSC retain their neuroprotective potential *in vivo* we employed retinal ischemia/reperfusion (I/R) as a model of central nervous system (CNS) injury (Fig. 4A). As expected, 1 hour of ischemia induced significant retinal damage as measured by retinal ganglion cell (RGC) loss. Eyes in the vehicle-only control group displayed a loss of approx. 88% of RGC seven days after I/R injury when compared to the non-ischemic contralateral eyes (I/R + PBS: 309 ± 308 RGC mm^2^; non-ischemic: 2413 ± 413 RGC mm^2^; p < 0.001, Fig. 4B,C). Loss of RGC was significantly ameliorated in the presence of fresh MSC. Transplantation of these cells resulted in survival of 829 ± 405 RGC mm^2^ (p = 0.0019). Moreover, transplantation of cryo-MSC provided an equivalent effect on RGC survival (845 ± 320 RGC mm^2^) and is also statistically significant when compared to the RGC density of the vehicle group (p = 0.024).

**Figure 5:**
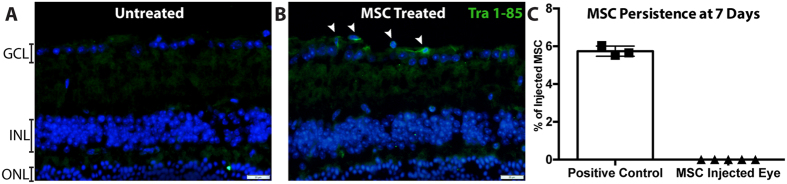
Cryopreserved MSC do not persist in the eye following ischemia/reperfusion injury. Human cells, identified by human specific Tra 1–85 immunostaining and counterstained with DAPI were undetectable in (**A**) untreated eyes and rare in (**B**) treated eyes at 3 days, white arrows indicate positive cells atop the ganglion cell layer. (**C**) Quantitative PCR for human genomic DNA remaining in mouse retina seven days after transplantation. Human DNA was not detected in any of the tested eyes (n = 5, mean ± SD). Data normalized as % of injected as 30,000 MSC were delivered to each eye. Donor 7083 used for all mice. Positive control is DNA extracted from 1,670 MSC, corresponding to 5.6% of the total injected MSC). GCL: Ganglion cell layer, INL: Inner Nuclear Layer, ONL: Outer Nuclear Layer.

Rescue of RGC did not appear to depend on engraftment or persistence of MSC in the eye. Staining the human cell surface antigen Tra 1–85 has been used successfully to identify human cells, including MSC, in xenogenic transplant experiments^20^,^21^. By staining retina sections for Tra 1–85, a small portion of surviving MSC could be consistently detected in I/R eyes three days after transplantation (Fig. 5A,B). The remnant of MSC were found either in the vitreous, on the surface of the nerve fiber layer, or partially integrated, in the retinal ganglion cell layer (Fig. 5B). After 7 days, no MSCs were detected and furthermore, no signs of tumor formation were observed in any of the eyes by histological analysis (n = 7). To confirm MSC did not persist in the eye and no human derived tumors had formed, RT-PCR was performed. PCR amplification of genomic DNA extracted from the retinas of treated eyes did not indicate the presence of human DNA within the mouse tissue 7 days after transplantation (Fig. 5C). These data indicate that all of the tested samples contained fewer than 17 cells, which was the lowest concentration included in the standard curve, and support our immunohistochemical findings suggesting that MSC do not persist in the retina at 7 days. The absence of human genomic DNA also demonstrates the absence of MSC derived tumors. Thus our data not only demonstrate that use of human MSC is safe and effective in this mouse model of retinal I/R injury but are also congruent with our *in vitro* data indicating that cryopreservation does not significantly diminish the function of MSC.

## Discussion

Successful application of MSC to treat numerous conditions in animal models has led to a rapid increase in clinical trials exploring MSC therapy^2^. The safety of MSC therapy in clinical trials to date has only hastened the exploration of MSC for more and more conditions^22^. While most studies using animal models and even small clinical trials have utilized fresh MSC cultured on-site, cryopreservation of MSC is essential to the widespread application of MSC-based therapies. Cryopreservation allows for MSC to be prepared by specialized facilities, in large batches under the application of accepted quality control measures. Preservation and storage is already routine for other tissue engineered products: Organogenesis’ Apligraf can be stored for 15 days at 20–23 °C and Orthofix’s Trinity Elite bone allograft can be stored for weeks at −80 °C allowing off-the-shelf use as the need arises and eliminating the need for on-site GMP cell culture facilities.

Importantly, the availability of cryo-MSC enables administration of large doses of cells without time delay caused by culture expansion. While development of cryopreservation techniques for MSC that minimize loss of therapeutic function is a critical step to advancing MSC-therapy for all applications, many acute-onset conditions such as ischemic events or acute GvHD would specifically benefit from an off-the-shelf MSC therapy. Tissue damage in these conditions is often rapid and without immediate treatment, the desired therapeutic effect may not be fully realized. For example, in the retinal ischemia/reperfusion model employed herein almost all damage occurs within the first 72 hours after the insult and little additional damage is observed 7 days after reperfusion^23^.

To date, studies focused on the impact of cryopreservation on MSC function have yielded mixed results^24^. Cryopreservation of MSC has become routine and MSC stored for extended periods of time have been shown to have low tumourigenic potential^25^, maintain growth kinetics upon thawing^26^, and remain capable of multilineage differentiation^26^,^27^,^28^,^29^. When MSC were examined for their ability to differentiate to form bone, cryopreservation did not significantly impact the differentiation capacity of the cells in *in vitro* assays^26^ or after *in vivo* transplantation^27^,^28^,^29^. Subcutaneously implanted scaffolds in primate^27^ and murine^28^,^29^ models have revealed no statistical difference between fresh and cryo-MSC’s osteogenic potential. In contrast, studies focused on the use of MSC for their secreted trophic factors have reported detrimental effects from cryopreservation. Most notably, MSC have been reported to have poor viability after cryopreservation which reduces the number of cells capable of responding to inflammatory cues^7^. In addition, cellular debris in one study induced hyperproliferation of T-cells in co-culture experiments^7^. While MSC function in *in vitro* potency assays are informative, the critical question is whether cryopreservation impairs MSC function *in vivo*. Until now, data comparing the efficacy of cryo-MSC compared to fresh MSC *in vivo* and in humans has been limited. A post-hoc analysis of clinical outcomes in acute GvHD patients receiving intravenous injection of fresh or cryo-MSC revealed patients receiving fresh MSC tended to respond better than patients receiving cryo-MSC^9^.

We sought to determine whether cryopreservation would impact the therapeutic potency of MSC intended to combat I/R injury. Multiple mechanisms lead to cell death in the context of ischemia/reperfusion injury in the CNS. In addition to hypoxic insult, reperfusion of the ischemic tissue results in the generation of damaging reactive oxygen species and an influx of inflammatory cells that cause sterile inflammation^30^. While inflammation benefits clearance of damaged cells, activated neutrophils in sterile inflammation often contribute to damage, and viable cells are indiscriminately killed while cellular debris is cleared. Sterile inflammation has been well documented to significantly contribute to tissue damage following ischemic events in a variety of tissues^31^. For example, inhibition of neutrophil function by knocking out Nlrp3 prevented inflammosome activation and significantly reduced the extent of tissue damage in a model of acute renal ischemia^32^. In addition, antigen presenting cells such as dendritic cells are exposed to antigens from dying cells and damage associated molecular patterns (DAMPs) released by necrotic cells that serve as adjuvants^33^. These activated dendritic cells can then migrate to lymph nodes where they stimulate a T-cell response to antigens in the ischemic tissue^34^. Thus, preservation of both cell viability and MSC immunomodulatory properties is critical to the successful use of cryo-MSC to treat ischemia/reperfusion injury.

Numerous cryopreservation techniques and cryoprotectants have been used to preserve MSC with variable effects on the phenotype of cells post-thaw. Recent reviews by Yong *et al.*^24^ and Marquez-Curtis *et al.*^35^ provide an in depth overview of emerging cryopreservation technology. As viability of cells post-thaw appeared to be a predictor of MSC function in past studies, we used a cryopreservation media and method that has worked well in our lab and others^36^ for maximizing post-thaw recovery. MSC were frozen in serum-free, xeno-free CryoStor CS5 media containing 5% DMSO at a concentration of 1 × 10^6^/ml using a controlled-rate freezing cell at 1 °C/min. This process consistently yields MSC with >95% viability post-thaw. Cryo-MSC had an overall similar profile of secreted growth factors compared to passage and donor matched fresh MSC. MSC preserved and thawed using our protocol responded to exposure to IFN-γ by synthesizing IDO and converting tryptophan to kynurenine at the same rate as fresh MSC. In addition, these cryo-MSC remained suppressive of anti-CD3/anti-CD28 activated PBMCs in co-culture assays. While these findings stand in contrast to previous studies that reported impaired immunosuppressive properties using cryopreservation techniques that yielded 40–50% dead cells after thawing^7^, they are consistent with the notion that post-thaw viability is critical for post-thaw immunomodulatory function. Thus, viability, and not cryopreservation per se, appears to be a predictor of MSC function. Our study demonstrates that when viability is maintained, MSC remain functional in *in vitro* potency assays regardless if they are fresh or cryopreserved. This is in agreement with a recent study from RoosterBio which reported cryopreservation in CryoStor CS5 results in MSC with high viability, IDO activity, and similar cytokine secretion compared to fresh MSC^36^.

Finally, we demonstrated cryo-MSC remain therapeutic when injected immediately after thawing in a retinal model of I/R. Retinal I/R leads to rapid destruction of RGCs, neurons on the surface of the retina that transmit signals received from photoreceptor cells via bipolar and amacrine cells to visual processing centers of the brain through long axons that form the optic nerve^37^. The inner layer of the retina, were the RGC cell bodies reside, have been demonstrated to be particularly sensitive to hypoxia^38^, thus the ability to salvage RGC in the setting of I/R is critical if some degree of vision is to be preserved. In addition, retinal I/R injury not only resembles various ophthalmologic disorders causing visual impairment such as ischemic optic neuropathies and glaucoma, but also recapitulates many aspects of CNS injury, in particular stroke^39^,^40^,^41^. The analogy in the pathobiology with respect to hypoxia, oxidative stress and inflammation makes the animal model of retinal I/R extremely suitable to determine whether transplantation of cryo-MSC into the ischemic retina improves RGC survival as an off-the-shelf therapy. The rapid onset of reactive oxygen species and inflammatory cells in the ischemic tissue following reperfusion requires any mitigating treatment to be applied quickly if ganglion cells are to be salvaged. Thus, cell therapy strategies that require *in vitro* culture prior to infusion are not suitable for treating I/R injury. In our model, we demonstrated that MSC could be taken directly from cryostorage, thawed, washed, and injected into the ischemic tissue without impairing the therapeutic potency of the MSC. These findings are similar to those of earlier studies that suggested that retinal transplantation of MSC improves RGC survival after injury^16^,^42^. However, these studies relied on experimental designs that employed fresh MSC transplanted prior to injury. Herein we significantly extend these findings by demonstrating that transplantation of human MSC taken directly from cryostorage and injected two hours after reperfusion is significantly protective. This is a crucial difference from a translational point of view as our model recapitulates a clinical scenario, in which patients seek treatment in the hours following an ischemic event, receive reperfusion therapy, and would then be able to receive an off-the-shelf therapy to prevent secondary damage from the I/R injury. Our finding that MSC can be cryopreserved without an apparent loss in efficacy suggests that storage and use of MSC in a clinical setting may be feasible for I/R injury to the eye and CNS.

The success of MSC clinical therapy will ultimately depend on the availability of a rapidly accessible source of reliably effective cells. Cryopreservation greatly simplifies the logistics of cell therapy, by allowing centralized GMP facilities to grow and phenotype MSC. In addition, cryopreservation allows MSC to be used on-demand, eliminating the need to wait for cells to be expanded or acclimated in culture prior to use. However, cryopreserved cells only have therapeutic utility if their potency is preserved in the process. The work presented here outlines a simple and effective method for cryopreserving MSC that maintains >95% viability, expression of immunomodulatory factors and growth factors, and the ability of MSC to suppress activated immune cells. In addition, we demonstrate cryo-MSC perform as well as fresh MSC in a retinal model of I/R injury. Thus, we observed no major detriment in MSC phenotype or potency in *in vitro* and *in vivo* assays following cryopreservation making cryo-MSC a feasible off-the-shelf therapy for some indications. Further studies are warranted targeting additional disease indications and delivery routes to fully elucidate conditions and modes of delivery that are compatible with freshly thawed cryo-MSC.

## Materials and Methods

### MSC Cultures and Cryopreservation

Pre-characterized human MSC were provided by the Texas A&M Health Science Center College of Medicine Institute for Regenerative Medicine at Scott & White through a grant from NCRR of the NIH, Grant # P40OD011050, resource ID SCR_005522. Two donors of MSC, #7083 and #8002L, were obtained from Texas A&M and both were accompanied by a complete analysis of MSC surface marker expression and differentiation capacity in accordance with the ISCT minimal criteria for MSC^43^. Specifically, MSC from both donors were >95% positive for CD73a, CD90, and CD105, <2% positive for CD11, CD14, CD19, CD34, CD45, CD79a, and HLA-II, and capable of multilineage differentiation. MSC were plated at a density of 5,000 cells/cm^2^ in MEM-alpha supplemented with 15% fetal bovine serum (Premium Select, Atlanta Biologicals), 1% penicillin/streptomycin, and 1% L-glutamine unless otherwise stated. Cells were passaged when cells reached 70–80% confluence, which typically corresponded to 2.5 population doublings. All MSC were between population doubling levels of 6–11 at the time of use (Passage 3–5). MSC were cryopreserved using CryoStor CS5 cryopreservation media (Sigma, St. Louis, MO) and a CoolCell controlled rate freezing container (Biocision, San Rafael, CA). Briefly, MSC were harvested from cultures, pelleted, and resuspended in 4 °C Cryostor CS5 at a concentration of 1 × 10^6^/ml and aliquoted into cryovials. Vials were then placed in a CoolCell pre-chilled to 4 °C and placed in a −80 °C freezer for at least 90 minutes. Vials were then rapidly transferred to a pre-chilled liquid nitrogen storage box and maintained in liquid nitrogen vapor for 7–30 days before thawing. For thawing, all vials were removed from liquid nitrogen and placed directly in a 37 °C water bath until a small ice pellet remained. Cells were then gently pipetted into 4 ml pre-warmed media, centrifuged at 500 × g for 5 min and resuspended in 1 ml pre-warmed media for use in downstream applications. For *in vivo* transplantation, cryopreserved MSC (cryo-MSC) were thawed, resuspended in room temperature PBS−/− (without calcium and magnesium), counted, centrifuged, and resuspended in ice cold PBS−/− at a concentration of 10 × 10^6^/ml and placed on ice. All intraocular MSC injections occurred within 1 hour of thawing. ‘Fresh’ MSC in this study were all maintained in culture for at least 7 days. Cryo-MSC were used immediately after thawing unless otherwise noted. All experiments used donor and passage matched MSC to isolate the effect of cryopreservation on MSC phenotype and function.

### Viability and Metabolic Activity Assays

For viability analysis, terminal deoxynucleotidyl transferase dUTP nick end labeling (TUNEL) staining was performed on fresh MSC and MSC thawed directly from cryopreservation. In both cases, cells were washed twice, resuspended in PBS, and either analyzed immediately or after 1 hour of storage on wet ice. TUNEL staining was performed using the Apo-Direct Apopotosis Detection Kit (BD Biosciences, Franklin Lakes, NJ) according to the manufacturer’s instructions. Briefly, cells were fixed in 2.5% neutral buffered formalin (Sigma, St. Louis, MO) for 60 minutes, washed three times, fixed, and permeabilized in 70% ethanol at −20 °C for 3 days. Post thawing, double strand breaks were stained by incubation with FITC-dUTP followed by staining of all nuclei with propidium iodide (PI). The samples were analyzed by an Accuri C6 flow cytometer, with positive and negative control cells used for gating and color compensation. Fluorescent images of all samples were also acquired as validation. For viability analysis of fresh and cryo-MSC in the days after thawing, 30,000 MSC were seeded into a 24-well plate in triplicate for each experiment and cultured for 24, 48, and 72 hours. At each time point, media was removed from wells and 200 μL of staining media containing Hoechst and PI (Invitrogen, Carlsbad, CA) was added to each well. After 20 minutes of staining at 37 °C, four random fields were imaged to detect PI positive nuclei and total nuclei. Cells incubated with 1 μM staurosporine (Tocris, San Diego, CA) for 3 hours served as a dead control to verify the staining procedure and set the acquisition settings. Images were captured using an inverted phase contrast and fluorescent microscope with a 10x objective (DMI6000B, Leica Microsystems, Wetzlar, Germany). ImageJ (NIH) was used for nuclei counting.

Metabolic activity in the days following thawing was measured using an XTT assay (ATCC, Manassas, VA) after 24, 48, and 72 hours. Here, 15,000 MSC were placed in wells of a 96-well plate in 100 μL of culture media. Both fresh MSC, cultured for >7 days, and cryo-MSC were plated in duplicate or triplicate for each time point and metabolic activity was measured according to the manufacturer’s instructions. Wells with half the cell starting density with XTT, media only with XTT, and MSC alone without XTT were used as controls for each experiment (n = 4).

### Growth Factor Array

Growth factor secretion from fresh and cryo-MSC was assessed using the Human Growth Factor Array Q1 (RayBiotech, Norcross, GA). Here, either 200,000 fresh or cryo-MSC were cultured in 2 mL of 1% (v/v) FBS, 1% (v/v) Penicillin/Streptomycin, and 1% (v/v) L-glutamine supplemented MEM-α media in T25 flasks for 48 hours with or without 100 ng/mL human IFN-γ (PeproTech, Rocky Hill, NJ) and 50 ng/mL human TNF-α (Invitrogen, Carlsbad, CA). Media was collected and frozen at −20 °C along with cell lysate for western blot and IDO activity analysis. The array was performed per the manufacturer’s instructions using the growth factor standards provided in the kit and the media was loaded into the array with no dilution and at a 4x dilution. The slide was read and data extracted by RayBiotech. The total concentration of each growth factor in basal media and MSC conditioned media was interpolated using the standard curves for each factor. To determine growth factor concentration contributed by the MSC, the concentration of growth factor in the basal media was subtracted from the concentration measured in the MSC conditioned media.

### Western Blot

Cell lysates were collected from MSC by washing T25 plates in chilled PBS three times to remove media followed by addition of 80 μL chilled RIPA buffer with protease inhibitor cocktail (Santa Cruz Biotechnology, item# sc-24948A) and agitation with a cell scrapper. Tubes were incubated on ice for 15 minutes and lysate was clarified by pelleting precipitate at 8,000 × g at 4 °C for 10 minutes. Prior to loading, total protein content was measured by microBCA (Thermo Scientific, Waltham, MA). 10–20 μg of protein was loaded into each well of a precast Bolt 4–12% Bis-Tris gels. After transfer, the membranes were blocked with 5% non-fat dry milk and stained with their respective primary antibodies (1:1000 rabbit anti-IDO (12006S, Cell Signaling, Danvers, MA), 1:20,000 mouse anti-β-actin (1406030, Ambion, Thermo Scientific, Waltham, MA)). Horseradish peroxidase conjugated antibodies (1:10,000 goat anti-rabbit (A2315), 1:10,000 goat anti-mouse (H2014), Santa Cruz Biotechnology, Dallas, TX) were used as a secondary followed by incubation with SuperSignal West Femto chemiluminescent substrate (Thermo Scientific, Waltham, MA). Western blot images were visualized using an Odyssey C-Digit scanner and processed using Image Studio software (LI-COR Biosciences, Lincoln, NE). All Westerns were repeated 2–3 times and representative blots are displayed. Full length blots are provided in Supplemental Information.

### IDO Activity Assay

Media collected from fresh or cryo-MSC stimulated with or without human IFN-γ/TNF-α was analyzed for kynurenine content as a marker of IDO activity^44^. L-kynurenine (Sigma Aldrich, St. Louis, MO) was dissolved in culture media and used to create a standard curve. 100 μL of conditioned media or standards were placed in a 96-well plate. 50 μL of 30% (w/v) trichloroacetic acid was added to each well to precipitate out proteins. The plate was heated for 30 minutes at 52 °C to facilitate the conversion of N-formylkynurenine to kynurenine and then centrifuged at 1,200 × g for 15 minutes. 75 μL of supernatant from each sample or standard was mixed with 75 μL of Ehrlich’s reagent (0.8% (w/v) 4-(Dimethylamino)benzaldehyde in acetic acid), and incubated at room temperature for 10 minutes. The plate was then read at 492 nm and the concentration of kynurenine in each sample was interpolated from the standard curve.

### PBMC Co-cultures

MSC immunosuppressive capability was assessed by direct co-culture with isolated PBMCs from leukapheresis reduction cones obtained from the DeGowin Blood Center at the University of Iowa Hospital and Clinics. MSC to PBMC ratios (1:3, 1:6, and 1:12) were established by seeding 250,000 PBMCs in wells containing 83,300 MSC, 41,600 MSC, or 20,800 MSC of a 48-well plate. MSC were plated 1 hour prior to addition of PBMCs in RPMI supplemented with 10% (v/v) FBS, 1% (v/v) Penicillin/Streptomycin, and 1% (v/v) L-glutamine. PBMCs were labeled with CellTrace CFSE Cell Proliferation Kit (Invitrogen, Carlsbad, CA) at a final dye concentration of 1 μM. The PBMCs were then stimulated with 250,000 Human T-activator CD3+/D28+ Dynabeads (Invitrogen, Carlsbad, CA) in each well and cultured for 6 days. PBMC only with or without Dynabeads served as activated and un-activated controls respectively for all experiments. After 6 days, PBMCs were dispersed by gentle pipetting, collected, centrifuged at 500 × g for 5 minutes, and resuspended in 100 μL RPMI before analysis on an Accuri C6 flow cytometer. Unstimulated control PBMCs were used to set the gating threshold for each experiment (n = 4).

### Retinal Ischemia/Reperfusion Injury and MSC Transplantation

All animal experiments were carried out in accordance with the ARVO Statement for the Use of Animals in Ophthalmology and Vision Research and were approved by the IACUC committee of the University of Iowa. Unilateral retinal damage was induced by Ischemia/Reperfusion (I/R) injury as described earlier^23^,^45^,^46^. Briefly, male and female two-month old C57BL6/J (The Jackson Laboratory, Bar Harbor, ME) were anaesthetized by intraperitoneal injection of Xylazin/Ketamine (10 mg/kg and 100 mg/kg, respectively). Eyes received 0.5% proparacaine eye drops for topical analgesia, pupils were dilated with 0.5% tropicamide (both Akorn, Lake Forest, IL), and corneas were kept moist until animals had fully recovered (GenTeal, Alcon, Fort Worth, TX). The anterior chamber was cannulated with a sterile 30-gauge needle, which was connected to a saline reservoir by a perfusion line. Intraocular pressure (IOP) was elevated to 80 mmHg in left eyes by setting the saline reservoir to an equivalent height (108 cm) above the mouse’s head. Retinal ischemia was confirmed by blanching of the fundus and stasis within retinal vessels using fundus imaging. After one hour of IOP elevation, the cannula was carefully removed and reperfusion was evident by resumption of retinal blood and recovery of pulsation.

Two hours after I/R, animals underwent isoflurane sedation and I/R eyes were treated with either fresh MSC (I/R + Fresh MSC, N = 10) or cryo-MSC (I/R + Cryo-MSC, N = 17). 3 × 10^4^ MSC in 3 μl PBS were transplanted into the vitreous cavity using a Hamilton syringe equipped with a 33-gauge needle. A vehicle control group received an equivalent volume of PBS (I/R + PBS, N = 10). The right eyes of all mice received no manipulation and served as controls. Animals were euthanized at either three or seven days after I/R injury by CO_2_ inhalation followed by cervical dislocation.

### Detection of transplanted MSC

Eyes of animals having received cryopreserved MSC were harvest three (N = 5) days after transplantation, fixed in 4% paraformaldehyde for 2 hours, sucrose embedded and processed for traversal sectioning. Eyes (N = 7) obtained seven days after MSC transplantation were spilt in half; DNA was extracted from freshly isolated retinas for RT-PRC experiments from one half of the eye while the other half was processed for immunostaining. 7 μm sections were blocked in 1% BSA/PBS for 30 min, washed and incubated with goat anti-human Tra 1–85 antibodies (1:50 in 0.3% Triton X-100/PBS, R&D Systems, Minneapolis, MN) overnight. Slides were washed with PBS and an Alexa Flour 488 donkey anti-goat secondary antibodies (1:400 in PBS) was applied for 3 hours. After final rinses, nuclei were visualized using DAPI. Sections were coversliped and images were taken using an Olympus BX41 microscope.

### Detection of human DNA

Genomic DNA was extracted from the posterior retina of five eyes seven days after MSC transplant using DNeasy columns (Qiagen, Valencia, CA). 250 ng of this DNA was used in a quantitative PCR reaction using primers specific for human genomic DNA (forward: GAGAGCGTTTGGAAATTGGA, Reverse: TGGCTGCTGTTTCATGTCTC). Samples were amplified in a quantitative PCR reaction for 45 cycles using a CFX96 thermal cycler (BioRad, Hercules, CA). Data were compared against a standard curve was constructed using genomic DNA extracted from a known quantity of human MSC (17 to 16,750 cells). A positive control containing DNA from 1,675 MSC and a negative control (water only) were included. All measurements were taken in triplicate.

### Analysis of Retinal Ganglion Cell Survival

All animals were euthanized seven days after I/R injury by CO_2_ inhalation followed by cervical dislocation. Eyes were enucleated and fixed in 4% paraformaldehyde for 2 hrs. As previously described^47^,^48^, retinas were immunostained for γ-synuclein, a marker for retinal ganglion cells (RGC), and the number of surviving RGC was determined. Briefly, retinas where incubated overnight with mouse anti-γ-synuclein primary antibody solution (1:400, Abnova Corporation, Walnut, CA, USA), followed by several rinses in PBS and incubation with an Alexa Fluor 488 donkey anti-mouse secondary antibody (1:300, Life technologies, Grand Island, NY). After another PBS wash, retinas were whole-mounted, cover slipped and imaged. Twelve images (318 × 318 μm, 40X magnifications) were taken at predetermined mid-peripheral locations using a Nikon Eclipse i80 confocal microscope (Nikon Instruments Inc, Melville, NY). γ-synuclein positive RGC were counted in a masked fashion by an independent observer using the cell counter plugin in ImageJ software (NIH).

### Statistical Analysis

For statistical comparisons *in vitro* between fresh and cryo-MSC, One-way ANOVA with Sidak correction for multiple comparisons with significance set at p < 0.05 was used in Prism 6 (GraphPad, San Diego, CA). For *in vivo* experiments, averaged RGC data was analyzed using Tukey’s honest significant difference (HSD) post hoc tests (with unequal *N*) in Statistica software (Dell, Round Rock, TX). P-values < 0.05 are considered as statistically significant. All data are given as mean ± standard deviation (SD).

## Additional Information

**How to cite this article**: Gramlich, O. W. *et al.* Cryopreserved Mesenchymal Stromal Cells Maintain Potency in a Retinal Ischemia/Reperfusion Injury Model: Toward an off-the-shelf Therapy. *Sci. Rep.* **6**, 26463; doi: 10.1038/srep26463 (2016).

## Supplementary Material

Supplementary Information
