# Supplementary material for: Cryopreserved Mesenchymal Stromal Cells Maintain Potency in a Retinal Ischemia/Reperfusion Injury Model: Toward an off-the-shelf Therapy
Source: Sci Rep. 2016 May 23;6:26463. doi: 10.1038/srep26463 (PMC4876464; doi:10.1038/srep26463)
Supplement: Supplementary Information [file srep26463-s1.pdf]

Cryopreserved Mesenchymal Stromal Cells Maintain Potency in a Retinal Ischemia/Reperfusion Injury Model: Toward an off-the-shelf Therapy

Oliver W. Gramlich[1,2], Anthony J. Burand[3,4], Alex J. Brown[3,4], Riley J. Deutsch[3,4], Markus H. Kuehn[1,2], James A. Ankrum[3,4*]

[1]Department of Ophthalmology and Visual Sciences, University of Iowa, Iowa City, IA

[2]Center for the Prevention and Treatment of Visual Loss, Iowa City VA Health Care, Iowa City, IA

[3]Department of Biomedical Engineering, University of Iowa, Iowa City, IA

[4]Fraternal Order of Eagles Diabetes Research Center, Pappajohn Biomedical Institute, University of Iowa, Iowa City, IA

*Correspondence: James-ankrum@uiowa.edu

Table S1. Growth factor array data for 40 growth factors. For each condition, the concentration of the growth factor was calculated by subtracting the raw sample concentration from the media control baseline (i.e. [sample] = [raw sample] – [media control]). Thus concentrations in the table represent the mean±SD concentration of growth factor contributed by the presence of the MSC in pg/ml. Concentration values that were either below the media control baseline or the Assay Detection Limit (DL) are labeled '< DL'. If the media control was < DL, then no subtraction was performed on the sample concentration for the given growth factor. Each growth factor was measured 4 times and if 3 of the 4 measurements were below the detection limit, they were listed as '<DL'. For each growth factor, a one-way ANOVA with Sidak correction for multiple comparisons was performed to compare cryo-MSC to fresh MSC under stimulated and unstimulated conditions. A p-value <.05 was considered significant and multiplicity adjusted p-values are provided in the table.

Growth Factor	Media Control	Increase in concentration above media control: Unstimulated – Fresh MSC	Unstimulated – Cryo MSC	Fresh vs Cryo Unstimulated – Significant?	Fresh vs Cryo Unstimulated – p-value	Stimulated with IFN-γ/TNF-α – Fresh MSC	Stimulated with IFN-γ/TNF-α – Cryo MSC	Fresh vs Cryo +IFN-γ/TNF-α – Significant?	Fresh vs Cryo +IFN-γ/TNF-α – p-value	Assay DL (pg/mL)
**BMP-7**	< DL	< DL	295 ± 365	NA	-	< DL	474 ± 127	NA	-	54
**GDF-15**	< DL	24 ± 20	61 ± 17	Yes	0.01	48 ± 8	47 ± 13	No	0.99	3
**IGFBP-1**	8 ± 3	< DL	48 ± 10	NA	-	11 ± 12	< DL	NA	-	5
**IGFBP-2**	493 ± 40	821 ± 229	627 ± 291	No	0.32	324 ± 216	190 ± 174	No	0.68	27
**IGFBP-3**	< DL	1869 ± 130	1445 ± 41	No	0.47	714 ± 776	986 ± 381	No	0.75	270
**IGFBP-4**	1253 ± 477	159 ± 262	349 ± 264	No	0.39	< DL	< DL	NA	-	270
**IGFBP-6**	< DL	8563 ± 2102	9397± 1220	No	0.69	7870 ± 428	4931 ± 1663	Yes	0.03	135
**Insulin**	< DL	< DL	189 ± 123	NA	-	75 ± 139	34 ± 53	No	0.71	27
**OPG**	< DL	358 ± 31	594 ± 113	No	0.39	1386 ± 449	1540 ± 211	No	0.65	5
**PDGF-AA**	18 ± 7	11.6 ± 0.7	16 ± 7	No	0.43	7 ± 8	18 ± 2	Yes	0.04	13
**PIGF**	< DL	9 ± 5	8 ± 4	No	0.83	< DL	< DL	NA	-	5
**SCF R**	< DL	< DL	< DL	NA	-	67 ± 22	202 ± 155	No	0.06	27
**TGFb1**	< DL	< DL	< DL	NA	-	1403 ± 580	678 ± 584	Yes	0.05	135
**VEGF**	< DL	620 ± 210	796 ± 214	No	0.30	670 ± 120	703 ± 93	No	0.96	13
**AR**	< DL	< DL	< DL	NA	-	< DL	< DL	NA	-	13
**BDNF**	5 ± 2	< DL	< DL	NA	-	< DL	0.29 ± 0.08	NA	-	3
**bFGF**	420 ± 310	< DL	< DL	NA	-	< DL	< DL	NA	-	27
**BMP-4**	< DL	< DL	< DL	NA	-	< DL	< DL	NA	-	135
**BMP-5**	272 ± 57	< DL	< DL	NA	-	< DL	< DL	NA	-	135
**b-NGF**	< DL	< DL	< DL	NA	-	< DL	< DL	NA	-	13
**EGF**	< DL	< DL	< DL	NA	-	< DL	< DL	NA	-	0.3
**EGF R**	< DL	< DL	< DL	NA	-	< DL	< DL	NA	-	13
**EG-VEGF**	66 ± 51	< DL	< DL	NA	-	< DL	< DL	NA	-	13
**FGF-4**	862 ± 111	< DL	< DL	NA	-	< DL	< DL	NA	-	135
**FGF-7**	109 ± 16	< DL	57 ± 57	NA	-	< DL	< DL	NA	-	13
**GDNF**	29 ± 9	< DL	< DL	NA	-	< DL	< DL	NA	-	5
**GH**	170 ± 42	< DL	< DL	NA	-	< DL	< DL	NA	-	13
**HB-EGF**	< DL	< DL	< DL	NA	-	< DL	< DL	NA	-	13
**HGF**	39 ± 20	< DL	< DL	NA	-	< DL	< DL	NA	-	5
**IGF-I**	< DL	< DL	< DL	NA	-	< DL	< DL	NA	-	27
**MCF R**	< DL	< DL	< DL	NA	-	< DL	< DL	NA	-	54
**NGF R**	35 ± 23	< DL	4 ± 4	NA	-	< DL	< DL	NA	-	13
**NT-3**	< DL	< DL	< DL	NA	-	< DL	< DL	NA	-	54
**NT-4**	< DL	< DL	72 ± 12	NA	-	< DL	< DL	NA	-	13
**SCF**	< DL	< DL	< DL	NA	-	< DL	< DL	NA	-	13
**TGFa**	< DL	< DL	< DL	NA	-	< DL	< DL	NA	-	13
**TGFb3**	< DL	< DL	< DL	NA	-	< DL	55 ± 9	NA	-	54
**VEGF R2**	< DL	< DL	< DL	NA	-	28 ± 24	< DL	NA	-	13
**VEGF R3**	< DL	< DL	< DL	NA	-	< DL	< DL	NA	-	54
**VEGF-D**	< DL	< DL	29 ± 13	NA	-	< DL	< DL	NA	-	27

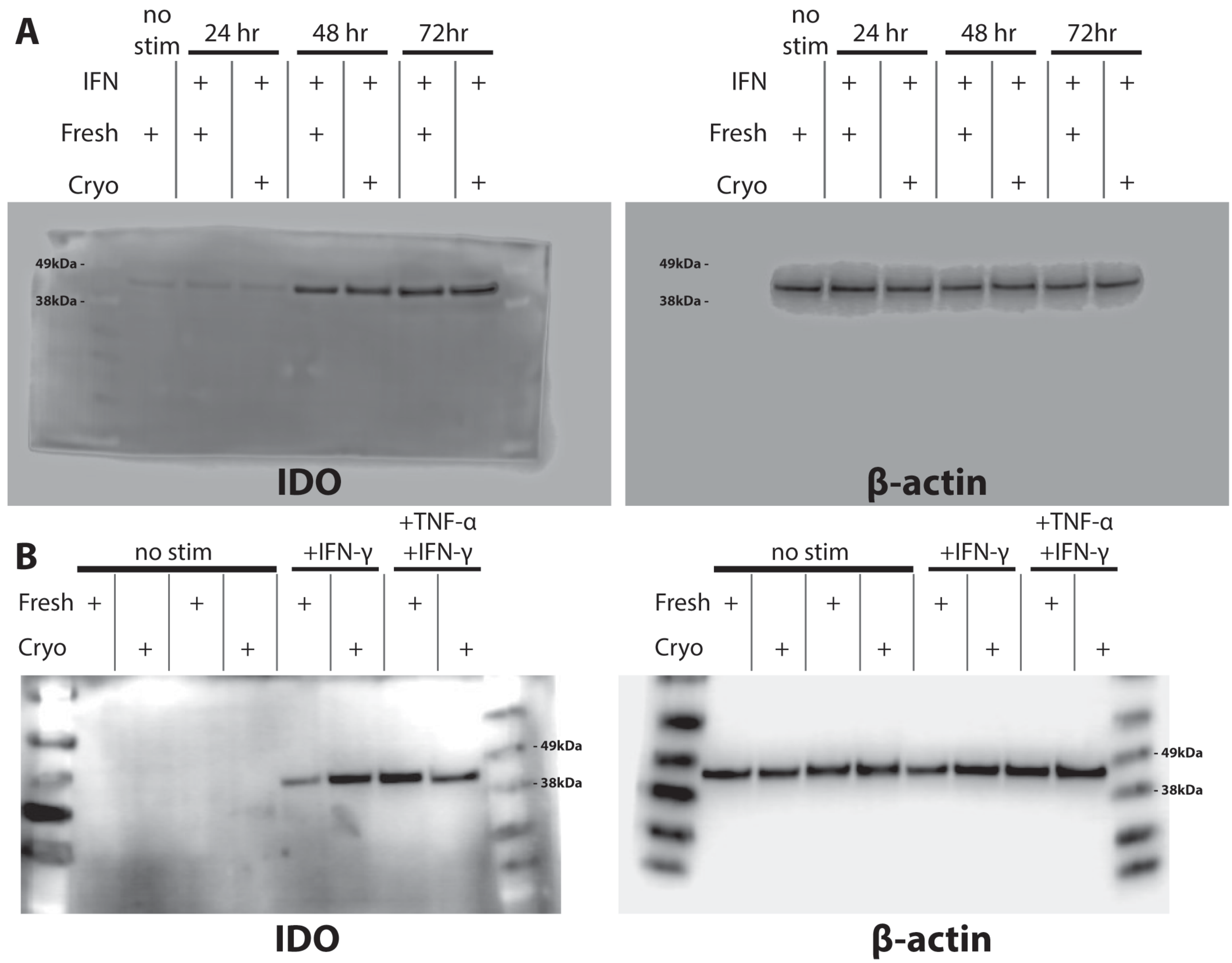

Figure S1. Full length blots from which the cropped blots in **(A)** Fig 2A and **(B)** Fig 2B were derived. Molecular weight markers were annotated based on the location of pre-stained molecular weight markers.
